# Does Risk Formulation Help Independent Review Board Decisions on Release of Prisoners? A Qualitative Study With Parole Board Members in England and Wales

**DOI:** 10.1002/cbm.70001

**Published:** 2025-07-16

**Authors:** Mary McMurran, Libby Payne, Alys Harrop, Nicola Bowes

**Affiliations:** ^1^ School of Sport & Health Sciences Cardiff Metropolitan University Cardiff UK; ^2^ Cardiff Metropolitan University Cardiff UK

## Abstract

**Background:**

The Parole Board for England & Wales makes decisions on the release or continued detention of people in prison. Psychological risk assessments (PRAs) assist in decision making and it is crucial that they are of good quality, including coherent and useful case formulations.

**Aims:**

The purpose of this study was to examine Parole Board members’ views on the accessibility, quality and usefulness of case formulations in PRAs.

**Method:**

Interviews were conducted with 8 psychologist/psychiatrist members and 11 independent/judicial members.

**Results:**

Respondents valued formulations in identifying idiosyncratic risk factors and linking these to risk management strategies. Nevertheless, they identified challenges to their validity, with concerns about facts versus hypotheses. Particular problems were seen in the assessment of those denying their offending and in collaborative case formulation. Integrating information and hypothesising under what conditions a risk factor might be activated was seen as important. Ignoring ethnic and cultural factors was seen as commonplace.

**Conclusion:**

The opinions of Parole Board users of PRAs provide information that could be used to improve the validity and usefulness of risk formulations, including adding to existing practice guidelines. A broader study of users’ perceptions of PRAs as a whole, not just formulations, would be useful and research on impacts is desirable.

## 1 Introduction

Current good practice in violence risk assessment is considered to be structured professional judgement (SPJ) using evidence‐based and validated risk assessment guidelines. Violence risk assessment guides, such as the Historical Clinical Risk Management‐20 (HCR‐20v3; Douglas et al. 2013) and the Sexual Violence Risk‐20 (SVR‐20v2; Boer et al. 2018), cover the individual's past experiences and behaviours, current presentation and issues to do with future management. The process includes gathering information from records and client interview and coding the existence and relevance of risk factors. This information then needs to be logically connected, based on theory and empirical evidence, into a formulation, which is a conceptually meaningful explanatory framework, based on postulating which scenarios for violence might be triggered in the future and what risk management strategies can be recommended (Douglas 2019; Hart and Logan 2011; Logan 2014). Logan (2014) suggests that, where possible, the formulation should also create a mutual understanding between evaluator and client, a point of some importance when those in prison may not understand their risk assessments and hence be unprepared to account for themselves at a parole hearing (Kelly et al. 2020).

The Parole Board for England and Wales makes decisions on the continuing detention or otherwise of people in prison on indeterminate sentences and certain determinate sentences. In many cases, psychological risk assessments (PRAs) are requested or submitted to assist a Parole Board panel in their decision making. These are part of the case dossier prepared for the panel. They may be commissioned by the Parole Board, by the person's legal representative, or both. All parties see the PRAs. Based on PRAs, psychologists make recommendations for release or continued detention and psychologists' opinions are highly influential in the parole process (Dyke et al. 2020). It is crucial that these PRAs are of good quality, including coherent and useful formulations. The purpose of this study is to examine Parole Board members' views on the accessibility, quality and usefulness specifically of formulations in PRAs thus to provide a basis for improvement.

## 2 Method

### 2.1 Participants

The aim was to recruit 12 psychologist or psychiatrist members and 12 independent (i.e., suitably experienced people from any background) or judicial members of the Parole Board for England and Wales. These numbers are based on evidence that virtually all themes in qualitative data are evident within a range of 6–17 interviews (Hennink and Kaiser 2022), and that, when focusing on a narrow topic in a highly specific sample, then small numbers give sufficient information power (Malterud et al. 2016). A list of current Parole Board members was obtained which listed 193 independent members and 25 judicial members (*N* = 218), and 51 psychologist members and 21 psychiatrist members (*N* = 72). Every 18th member in the independent/judicial list and every 6th member in the psychologist/psychiatrist list was selected to participate. If that person declined participation, the next person on the list was approached but with a time limited selection phase because of short‐term funding for a research assistant. Interviews were conducted with 8 psychologist/psychiatrist members (two psychiatrists; 7 women, 1 man; range of service length—3 to 14 years, Median = 4.5 years), and 11 independent/judicial members (one judge; 6 women, 5 men; range of service length—6 months to 17 years, Median = 5 years).

### 2.2 Materials

A semi‐structured interview (see Appendix 1) was constructed to tap into general matters of comprehensibility and usefulness but also to gather information relevant to published good practice guidelines (Hart et al. 2011; McMurran and Bruford 2016). It is acknowledged that one of the researchers in this study (MM) was a co‐author of these good practice guidelines.

### 2.3 Procedure

Ethical approval was obtained from Cardiff Metropolitan University (Reference STA‐9346) and approval was gained from the Parole Board's Research Governance Group. Participants were sent an information sheet and consent form prior to the first contact and time was offered to discuss these prior to the start of the interview. Interviews were conducted via an online video conferencing platform by a trained senior research assistant. Participants' interviews were audio‐recorded; video recording was optional. Agreement to proceed was taken as consent. Interviews took between 45 min and 1 h and participants were given £43 by the Parole Board in compensation for their time. Interviews were transcribed automatically using the platform's facility, checked for accuracy by the research assistant and the recordings were then deleted. Participants were shown the transcript and offered an opportunity to comment on or retract their interview. None did so.

### 2.4 Analysis

Narrative accounts were coded and thematically analysed deductively (Braun and Clarke 2006, 2022a). The focus was primarily on manifest meanings rather than underlying meanings because the questions were focused on specific pre‐determined aspects of formulation. MM constructed a code book for each group. We take the position that coding quality is not dependent on multiple coders (Braun and Clarke 2022b). Codes were collated into themes and sub‐themes (MM). These were reviewed for coherence and saturation through several iterations in collaboration with co‐researchers.

## 3 Results

Respondents acknowledged that parole panels put considerable weight on psychologists’ evidence and members valued psychologists' professional expertise. Formulation‐specific views are presented here, divided into themes and subthemes as shown in Table 1. Illustrative quotes are attributed by group membership, PP for psychologist/psychiatrist and IJ for independent/judicial.

**TABLE 1:** Themes and subthemes.

Theme	Sub‐theme
Improves understanding	Definition Clarity
Adds value	Usefulness Validity Focus
Critique of methods	Types of formulation Collaborative Presentation
Suggestions for improvement	Limitations Suggestions

### 3.1 Improves Understanding

#### 3.1.1 Definition

Newer IJ members were unclear about how to define formulation, nonetheless both they and more experienced IJ members identified the core meaning, using terms such as *a summary (IJ6), making sense of the index offence (IJ2)* and *understanding (IJ9).* There was a general awareness of the integration of historical information to explain the offending behaviour and identify risks leading to implications for future management. As expected, PP members were able to define formulation clearly. PP members were more likely to note that they would be theory and evidence based and they would include information about protective factors as well as risk factors.

#### 3.1.2 Clarity

All respondents were positive about the clarity of formulations. IJ members were familiar with the language but, in any case, technical terms were usually explained by report writers. Some specialist areas were less accessible, for instance, terms relating to autism and schemas.

### 3.2 Adds Value

#### 3.2.1 Usefulness

Formulations were considered useful in a number of ways. They not only identified risk factors but went beyond this to explain the links between background and offending, comment on the interplay of risk factors, inform intervention and management and give suggestions for how best to work with the person.

Definitely helps the panel with assessing things like the risk management plan against that formulation to see whether those factors are picked up and accounted for ….

IJ10

Formulations were seen as less helpful if they simply summarised the assessment.

I just don't think they are as meaningful and useful as they could be. …. Oftentimes … they don't add much, they're just a summary of what's already been written. And that's not what they're supposed to be ….

PP5

Value came from integration and interpretation.

It's helpful … to expand on not only this is a risk factor, but this is how and when this risk factor might come into play … somebody might, for example, be homeless, but the risk doesn't increase until such time that they are also associating with antisocial peers, and they're using a specific type of drug or alcohol that makes a difference.

PP6

Formulations were also valued as an indicator of the validity of the psychologist's report.

I think the formulation gives you a sense of how confident you are in that professional’s understanding of the case and … how much weight you've been able to put on their professional opinion and their recommendations.

PP3

However, formulations were considered less necessary for simpler cases, where they were seen as common sense. Indeed, respondents reported having their own formulations.

… sometimes it feels like it's just common sense. Like, if you've read what's come before, then the case formulation doesn't offer you any surprises … in the more complicated cases, then that's not true.

IJ1

#### 3.2.2 Validity

Questions arose as to the validity of some formulations, specifically those where the behaviour of interest was not currently observable in prison, such as child sex offending and intimate partner violence; where they were based on a proxy offence because of denial of the index offence; and where there has been a range of different offending behaviours. Explaining denial in terms of shame was questioned.

And sometimes you get … Oh, well, they can't talk about it because of shame. And it's like, well, you know, that's a bit simplistic … is that a generic ‘people find it difficult to talk about things because of shame’ or is that in this case specifically? And if so, why?

PP3

#### 3.2.3 Focus

There was a strong view that a focus on risk was what was required rather than a holistic approach.

If you can link the formulation to risk and then link that to the risk management plan … If you can tie all of that together, then I think that is helpful and can be part of your release decision and your rationale.

IJ9

A future focus was valued with comments on the impact of interventions, possible future risk scenarios and recommendations for risk management.

OK, so this is … going to happen, that might happen and what can we do about it?

IJ2

However, not all respondents were comfortable with speculation.

I could hypothesise til the cows come home with all of the guys that we're looking at, but I can't make a decision on that because it's not evidence. So yes, the facts are much more useful.

IJ3

### 3.3 Critique of Methods

#### 3.3.1 Collaborative

Formulations compiled in collaboration with the prisoner were seen to improve engagement and increase the prisoner's understanding of their behaviour. However, concerns were expressed about reliance on a prisoner's self‐report.

Sometimes you get collaborative formulations that are based on what the prisoner says about their offending, which can sometimes be very different to what the judge and CPS [Crown Prosecution Service] documentation and witnesses … said about the offending, and in those examples, the collaborative formulation isn't always that helpful because you're almost formulating something that's quite different from the problem.

PP3

#### 3.3.2 Types of Formulation

Most people were familiar with the 5P's model, namely Problem identification, Predisposing factors, Precipitating factors, Perpetuating factors, and Protective factors (Weerasekera 1993) and felt they did not need an explanation of it. Whereas this model was readily understood, some felt it could be reported in a simplistic fashion.

What you see a lot of is the … 5Ps formulation which is just essentially like the bullet point list … I find them the least helpful because it's just essentially listing issues that are sort of obvious to anyone from reading the documents.

PP3

Other models were less favoured. Compassion focused approaches were seen to have the potential to minimise risk.

It's all very positive, very compassion focused. … But I find those sometimes not very helpful because you're thinking, yeah, it could be the nicest guy in the world and he's really helpless, really engaging and he's really compliant but does he still want to rape a child?

IJ3

Formulations based on the Power Threat Meaning Framework (Johnstone et al. 2018) were seen not to focus on risk.

I really like the Power Threat Meaning way of formulating, but in my experience it's not particularly helpful when you're considering risk because … understanding someone and what possibly makes them function in the way they do doesn't always tell you how risky they are.

PP2

Desistance formulations were used, but infrequently. There was an observation that this was possibly just the inverse of a risk formulation.

I’m not really knowing … what that would tell me that would be different to what a risk formulation would tell me …..

PP5

#### 3.3.3 Presentation

A narrative presentation was preferred, telling a story.

The narrative form makes me more confident that the person has thought about what they're talking about.

IJ5

Bullet points were considered helpful for drawing attention to the main points but were not a substitute for the narrative.

…you can end up back at a list of risk factors …. Well, I could have … done that myself. … I need you to do the thinking for me about how this all pulls together.

PP6

Diagrams were liked by some but not others with a recognition that there were visual and verbal learners. Whereas some felt the diagram clarified matters, others were less convinced.

…it [the formulation] had lots of boxes with arrows and you could follow the boxes and the arrows … and you thought, well, this is amazing. It really helped you to go, ‘Oh, that goes there. All right’.

IJ7

However, diagrams can be over‐complicated and there is uncertainty about what boxes and arrows mean, especially when they are formatted differently (e.g., solid or dotted lines; curved or straight etc.).

… when I get a bad diagram and particularly the really complex ones with arrows and boxes all over the place, I don't even look at it because I get overwhelmed. … I don't find those ones with boxes and arrows at all helpful. I prefer words, yeah.

PP2

### 3.4 Suggestions for Improvement

#### 3.4.1 Limitations

The hypothetical nature of the formulation was mentioned.

It is usually explicitly stated this is a hypothesis … so if I feel that it's sort of going down very hard on one track from that hypothesis, I might ask, would you accept that there is another hypothesis ….

PP8

They can be over‐inclusive.

Some risk assessments … throw the entire kitchen sink into their formulation. So, it becomes almost like you can't really pinpoint what's really going on specifically for this person in their lives at this time. … you think well actually a lot of those would be risk factors for anyone at any time … there's too much information there and that kind of annoys me because it makes me feel, ‘Have you really thought this through?’

PP2

A level of understanding gained through assessment and formulation can obscure risk.

If you understand what makes someone behave in a particular way, and it's written in a sympathetic way, it can sometimes I think underestimate risk because you sort of understand why they're doing what they're doing.

PP2

Some important issues were seen to be bypassed. These included race and culture, and also issues that remained undetected because the psychologist does not have expertise in a particular area.

I think we have quite a problem with race … it's a taboo no one can mention it, so you will get an offender who is a Black man and … the entire trajectory is from experience of racism, of deprivation, of neglect, and it follows throughout his life …. If you read the formulation … you wouldn't know he's a Black man.

PP2

There was a concern that the relative weight put on various aspects of the assessment was unclear.

It's going to be quite tricky … for the author to pull together HCR‐20,[protective factors] and then case formulation and then put it forward into in terms of recommendations for future. And sometimes it's a little bit hard to see which part they've actually placed most weight on.

PP8

#### 3.4.2 Suggestions

There was a desire for brevity and avoidance of repetition. There was also a desire for consistency in presentation so that the reader knows what to look for and also how much weight to place on various aspects of the assessment.

It just would be really helpful if there's a little bit more consistency … and we could … really easily pull out what you need to pull out … and the weight of it. So, if that bit to the psychologist is the most important bit, then we need to kind of understand that I think.

IJ4

A statement about limitations was desirable.

What would be helpful is if a professional didn't think the formulation fits very well or has concerns about the gaps or the validity, that they draw those out and are upfront by saying …. This is a formulation, but there are still unexplained aspects or and if they've got any suggestions as to why that might be, that helps us as well.

IJ10

Guidance for production was suggested.

A push towards standardisation, probably using 5Ps model. I'm reluctant always to suggest that professionals should be constrained in any way … but in terms of the role in providing guidance to a parole panel, I think standardisation as much as possible would help … but we'll probably call it guidance.

PP8

## 4 Discussion

In the parole process, considerable weight is placed on psychologists' opinions; a view identified here as in earlier research (Shingler and Needs 2018). Imprisoned people too see psychologists as having power in the parole process (Shingler et al. 2020). Formulation is a key aspect of PRAs and it is important, therefore, to understand what users value and refine formulation to maximise that value. Parole decision making centres on public protection and so there is a need to focus on risk. Holistic case formulations are likely to be over‐general and distract from the primary issue. More clinical approaches, such as a compassion focus (Gilbert 2014) and the Power, Threat Meaning Framework (Johnstone et al. 2018), were not favoured for risk assessment, although they will undoubtedly have a role in other work.

A focus on risk requires clarity about what that risk is. Many of those in prison are versatile in their offending, meaning that they engage in a range of offence types. Parole members are required to focus on the likelihood of *serious* harm, and this should be reflected in formulations. Where the person being assessed denies or minimises their offending, this was considered to be a challenge to validity. While bearing in mind that denial is not in itself a barrier to a release decision, this is indisputable, and it speaks to the more general concern about formulations being hypotheses. Respondents saw a case for putting forward alternative hypotheses and also for stating limitations. This is recommended good practice for expert witnesses (Crown Office and Procurator Fiscal Service 2024). A further concern regarding validity arose from constructing formulations in collaboration with the person concerned. Information collected through self‐report may be inaccurate because of poor memory, lack of self‐knowledge, socially desirable responding and situational demands (Paulhus and Vazire 2007). However, the process of formulating engages the individual and self‐reported information can usefully be triangulated with recorded evidence, but care should be taken to clarify what is self‐report and to avoid reliance entirely on this.

There is a robust evidence base for risk factors for offending and, to a lesser degree, protective factors. Respondents preferred the 5Ps approach (Weerasekera 1993) as the framework for identification of these, but noted that simply listing all risk factors is unedifying, particularly without selective attention to the most potent factors. Also, what is needed is an account of the likely effects of interactions between important risk and protective factors (Craig et al. 2024). The identification of interaction effects is a difficult task, and it is perhaps here that it is necessary to single out the most potent risk and protective factors. Desistance formulations were frequently seen to be simply the inverse of risk formulations and so perhaps are redundant.

Overall, psychologists and psychiatrists were more concerned with the integration of information into a coherent and useful formulation, whereas the independent and judicial members were concerned about factual accuracy and questioned the hypothetical nature of hypotheses.

An important issue raised related to omissions and biases when formulating risk. Sometimes matters relevant to risk factors may be missed because psychologists will not have expertise in all areas (e.g., autism spectrum), and sometimes important social and cultural issues may be overlooked (e.g., experience of racism). The theoretical approach taken will also impact on the formulation (Eells and Lombart 2011). Different perspectives can be introduced through regular supervision or peer discussions to broaden the professional's scope.

Respondents understood the purpose and practice of formulation and felt able to produce a formulation for themselves, particularly for cases perceived as simpler. So, in resource terms, they need to be produced only for cases perceived as more complex. This has implications for parole panel chairs who request reports, as well as for providers.

Preference was generally for a narrative format, but bullet points and diagrams could be useful as summaries. A good narrative formulation was taken to be an indicator of how well the psychologist understood the person facing parole and consequently parole members could gauge how much weight to place on the psychologist's recommendations.

Diagrammatic representations of links between events and behaviours may not be consistent and could benefit from standardisation of format and include a legend indicating what various boxes, arrows and lines represent.

The main limitations of this study relate to the narrow focus and the researchers' background. The focus on formulation specifically omitted opinions on the PRA in its entirety, including recommendations. Three of the researchers (M.M., L.P. and N.B.) are forensic psychologists with experience of risk assessment and parole decision‐making. The interviewer (A.H.) was familiar with the principles of formulation but not forensic risk assessment. Interviews were therefore not biased by pre‐formed opinions of formulations, but analysis arguably benefited from forensic experience.

In conclusion, the opinions of users of PRAs provide useful information about how the focus, construction, and presentation of formulations could be developed to maximise usefulness. This information could be used to further develop practice guidance (e.g., guidance for prison and probation service psychologists). Further research could focus more broadly on users’ perceptions of PRAs. Evaluation of risk formulation in terms of impact would add to a sparse evidence base (Tarpey et al. 2023; Wheable and Davies 2020). For example, in a rare study of outcomes, Wheable et al. (2024) examined the impact of formulation‐based recommendations on forensic outcomes, such as prison recalls and licence breaches. There is considerable scope for examining the connections between formulation, recommendations and outcomes. This should include research on the direct impact on those whose risk is formulated as well as on professionals who use them.

## Data Availability

The data that support the findings of this study are available from the corresponding author upon reasonable request.

## Appendix 1 Semi‐Structured Interview

1. What do you understand a case formulation to be?
2. How easy are case formulations to understand (e.g., logical, explanatory and jargon free)?
3. What are your views on the amount of information usually included in case formulations (i.e., not enough information, too much information or is it usually just right)?
4. What are your overall views on the integration of the relevant information into a case formulation (i.e., is it usually coherent, understandable and helpful)?
5. Do you think case formulations add value over and above the identification of specific risk factors (e.g., substance use, problems managing emotions and antisocial attitudes)?
6. How useful do you find case formulations in the following ways?
  6a. How useful in making decisions about release, progress to less secure conditions or remaining in closed conditions?
  6b. How useful in terms of identifying appropriate interventions?
  6c. How useful in terms of identifying risk management strategies?
7. Have you seen different types of formulation (e.g., risk formulation, desistance formulation) (i.e., understanding the past vs. hypothesising about the future)?
  7a. If so, what are the differences in usefulness?
8. When psychologists make recommendations for interventions and management strategies to reduce risk, is it clear how are these linked to the case formulation?
9. Have you ever questioned a psychologist witness about the detail of their case formulation?
  9a. If so, what needed clarification?
10. What would make case formulations more useful to you for your Parole Board role?
11. Do you have any suggestions about the presentation of case formulations (e.g., place in reports or bullet points vs. narrative)?
12. Do you have any thoughts on how formulations fit with the decision‐making framework?
